# Racemization at the Asp 58 residue in αA‐crystallin from the lens of high myopic cataract patients

**DOI:** 10.1111/jcmm.13363

**Published:** 2017-10-10

**Authors:** Xiang‐jia Zhu, Ke‐ke Zhang, Wen‐wen He, Yu Du, Michelle Hooi, Yi Lu

**Affiliations:** ^1^ Eye Institute Eye and ENT Hospital Shanghai Medical College Fudan University Shanghai China; ^2^ Department of Ophthalmology Eye and ENT Hospital Shanghai Medical College Fudan University Shanghai China; ^3^ Key Laboratory of Myopia Ministry of Health PR China Shanghai China; ^4^ Shanghai Key Laboratory of Visual Impairment and Restoration Shanghai Medical College Fudan University Shanghai China; ^5^ University of Adelaide Adelaide SA Australia

**Keywords:** aspartyl residue, cataract, crystallin, high myopia, lens, racemization

## Abstract

Post‐translational modifications in lens proteins are key causal factors in cataract. As the most abundant post‐translational modification in the lens, racemization may be closely related to the pathogenesis of cataract. Racemization of αA‐crystallin, a crucial structural and heat shock protein in the human lens, could significantly influence its structure and function. In previous studies, elevated racemization from l‐Asp 58 to d‐isoAsp58 in αA‐crystallin has been found in age‐related cataract (ARC) lenses compared to normal aged human lenses. However, the role of racemization in high myopic cataract (HMC), which is characterized by an early onset of nuclear cataract, remains unknown. In the current study, apparently different from ARC, significantly increased racemization from l‐Asp 58 to d‐Asp 58 in αA‐crystallin was identified in HMC lenses. The average racemization rates for each Asp isoform were calculated in ARC and HMC group. In ARC patients, the conversion of l‐Asp 58 to d‐isoAsp 58, up to 31.89%, accounted for the main proportion in racemization, which was in accordance with the previous studies. However, in HMC lenses, the conversion of l‐Asp 58 to d‐Asp 58, as high as 35.44%, accounted for the largest proportion of racemization in αA‐crystallin. The different trend in the conversion of αA‐crystallin by racemization, especially the elevated level of d‐Asp 58 in HMC lenses, might prompt early cataractogenesis and a possible explanation of distinct phenotypes of cataract in HMC.

## Background

Cataract is a major cause of blindness worldwide. It is characterized by opacification and coloration in the centre of the lens and is accompanied by extensive post‐translational modifications (PTMs) in various proteins. Previous studies have revealed a relatively higher prevalence and earlier onset of nuclear cataract in patients with high myopia than in other patients 1. High myopia is more common in Asian populations than in other ethnic groups, and the proportion of HMC cases is increasing in Asian areas 2. However, the causal mechanisms explaining the early onset of cataract in this population remain unknown.

PTMs are closely related to lens opacification. A number of PTMs have been described in proteins isolated from relatively old human lenses, including deamidation, phosphorylation, methylation, truncation and racemization 3. According to previous studies, the racemization of amino acids, that is the conversion from the l‐ to d‐configuration is the most abundant modifications in long‐lived proteins 4. The normal l‐aspartyl (l‐Asp) residues are spontaneously converted to l‐isoAsp, d‐Asp and d‐isoAsp isomers *via* a succinimide intermediate, thereby generating all four Asp isomers in proteins 5.

Racemization of amino acids in long‐lived proteins occurs in various types of tissue in humans, including the lung, heart, brain, teeth, skin, intervertebral discs and cartilage 6. The most well‐known tissue with such long‐lived proteins is the lens. Owing to the high concentration of crystallins and lack of protein turn‐over, the lens is regarded as an ideal model for the study of protein PTMs 7. Previous studies have revealed that specific sites on lens proteins, such as alpha‐crystallin, are particularly prone to racemization 8. As an important structural protein in the lens, αA‐crystallin has long been a focus of studies on ARC formation and development. Truscott and colleagues identified significantly higher levels of d‐amino acids in ARC lenses than in age‐matched normal lenses, especially at Asp residues of crystallins 4. Asp 58 in αA‐crystallin from ARC lenses shows a relative acceleration of racemization compared with age‐matched normal lenses 9, indicating that it may be crucial for the induction of ARC and therefore is a critical residue to investigate thoroughly. These findings have been interpreted as evidence for the major quantitative significance of racemization in the age‐dependent denaturation and ageing process of lens proteins.

However, it remains unclear whether the racemization of αA‐crystallin plays a role in the pathogenesis of HMC. In this study, we extended the scope of racemization analysis to HMC. We examined racemization levels of αA‐crystallins in high myopia and evaluated variation in racemization at specific sites that may be associated with HMC, to improve our understanding of its role in HMC.

## Materials and methods

All tissues obtained during surgery were handled in accordance with the tenets of the *Declaration of Helsinki*. The Ethics Committees of the Eye and ENT Hospital, Fudan University, Shanghai, China and Guangming Hospital, Jinjiang, Jiangsu, China approved our collection and use of lens tissues from patients undergoing cataract surgery. Informed consent was obtained from all participants.

### Sample collection

Between 1 April 2015 and 1 October 2015, 6 ARC lenses and 6 HMC lenses were collected from patients undergoing cataract surgery. The HMC lenses and age‐matched ARC lenses were obtained from the Eye and ENT Hospital, Fudan University, Shanghai, China and Jinjiang Guangming Hospital, Jiangsu, China, during small‐incision cataract extraction. Before surgery, a thorough ophthalmic examination was performed. Cataract type and severity (NC: nuclear colour; NO: nuclear opacification) were graded according to the modified Lens Opacity Classification System III (LOCSIII). Patients with an axial length of ≥26 mm were diagnosed with high myopia. The preoperative exclusion criteria were as follows: a history of previous ocular surgery, glaucoma, uveitis and systemic diseases, such as diabetes mellitus. Six lenses with ARC and six lenses with HMC were used. For all lenses, the nucleus was separated from the cortex by coring through the visual axis with a 4.5‐mm‐diameter trephine, which was pre‐cooled at −20°C. Only the nuclear regions of adult human lenses were used for the analysis.

### Extraction and identification of αA‐crystallin

All samples were homogenized in lysis buffer (1 mM DTT, 150 mM Tris–HCl pH 8.0, protease inhibitor). The homogenate was sonicated on ice and clarified by centrifugation at 16000× ***g** *at 25°C for 10 min. Protein content was determined using BCA Protein Assay Reagent (Beyotime, Wuhan, China). The supernatants were considered the soluble proteins and stored at −80°C until use.

After isolating crystallin by gel filtration chromatography (using a Sephadex G‐200 Column) from the nuclear region of ARC and HMC lenses 10, 11, each component of various peaks from lens extracts was further confirmed and the existence of αA‐crystallin was verified by Western blotting. After determining the protein concentration using the Quick Start Bradford Protein Assay Kit (Bio‐Rad, Hercules, CA, USA), sample extracts were supplemented with 5× SDS‐PAGE loading buffer and denatured at 100°C for 5 min. Then, sample extracts were separated by 12% gradient SDS‐PAGE. Protein bands were transferred onto a PVDF blotting membrane (Millipore, Billerica, MA, USA) and subjected to immunolabelling using primary antibodies against CRYAA (1:1000 dilution; Abcam, Cambridge, MA, USA) and GAPDH (1:1000 dilution; Abcam). The membrane was incubated with a horseradish peroxidase (HRP)‐conjugated secondary antibody (1:2000; Abcam) for 30 min. at 25°C. Immunoblotted bands were revealed using the ECL Chemiluminescence Detection Kit (Amersham Pharmacia Biotech, Cleveland, OH, USA).

### Protein digestion

Protein digestion was performed according to the FASP procedure described by Wisniewski *et al*. 12, and the resulting peptide mixture was labelled using the 4‐plex iTRAQ reagent according to the manufacturer's instructions (Applied Biosystems, Waltham, MA, USA). Briefly, 200 μg of proteins for each sample was incorporated into 30 μl of SDT buffer (4% SDS, 100 mM DTT, 150 mM Tris‐HCl pH 8.0). The detergent, DTT and other low molecular weight components were removed using UA buffer (8 M Urea, 150 mM Tris‐HCl pH 8.0) by repeated ultrafiltration (Pall units, 10 kD). Then, 100 μl of 0.05 M iodoacetamide in UA buffer was added to block reduced cysteine residues, and the samples were incubated for 20 min. in darkness. The filters were washed with 100 μl of UA buffer three times and then 100 μl of DS buffer (50 mM triethylammonium bicarbonate, pH 8.5) twice. Finally, the protein suspensions were digested with 2 μg of trypsin (Promega, Madison, WI, USA) in 40 μl of DS buffer overnight at 37°C, and the resulting peptides were collected as a filtrate.

### Synthesis, purification and identification of peptides

The four isoforms of aspartate in TVLDSGISEVR, which correspond to tryptic peptide 55–65 of human αA‐crystallin with l‐isoAsp/d‐Asp/d‐isoAsp at position 58 9, were purchased from GL Biochem (Shanghai, China). After synthesized by reversed‐phase high‐performance liquid chromatography (HPLC) using a C18 column (GS‐120‐10‐C18‐AP, 30 × 250 mm, DiKMA, Beijing, China), peptides were purified by analytical HPLC using a C18 column (Kromasil, 4.6 × 250 mm, DiKMA) at a flow rate of 1 ml/min. and then identified by mass spectrometry (LCMS‐2000, Shimadzu, Kyoto, Japan).

### Racemization analysis of αA‐crystallin

Liquid chromatography (LC)–electrospray ionization (ESI) tandem mass spectrometry(MS/MS) analysis were performed on a LTQ‐Orbitrap Velos Pro mass spectrometer that was coupled to Easy‐nLC 1000 (Thermo Fisher Science, Odense, Denmark). Two microlitres of each fraction extracted from ARC and HMC lenses was injected for nano LC‐MS/MS analysis. Chromatography solvents were water (A) and acetonitrile (B), both with 0.1% formic acid. Peptide samples were concentrated and washed on an reverse phase trap column (75 μm × 2 cm; 5 μm; 100Å; C‐18, Thermo Scientific, Bremen, Germany) with 0.1% formic acid; then, they were eluted from the analytic column (75 μm × 15 cm; 3 μm; 100Å; C‐18, Thermo Scientific) with the following gradient 1–5% B (2 min.) At 41 min., the gradient increased to 20% B. At 51 min., the gradient increased to 40% B. At 53 min., the gradient increased to 100% B and was held there for 4 min. At 58 min., the gradient returned to 1% to re‐equilibrate the column for the next injection. Eluting peptides were directly analysed *via* tandem mass spectrometry (MS/MS) on an LTQ‐Orbitrap Velos Pro mass spectrometer (Thermo Scientific) equipped with a nano‐electrospray ion source.

A spray voltage of 1.8 kV and an ion transfer tube temperature of 250°C were applied. The instrument was calibrated before analysis using standard compounds and operated in the data‐dependent mode. The MS spectra was acquired in the m/z range of 160–1350, and survey scans were acquired in Orbitrap mass analyzer at a mass resolution of 60,000 at 400 m/z. Targeted peptides mass list of 588.32 acquired in the survey scans was chosen for CID fragmentation with normalized collision energy of 27%, and resolution for CID MS/MS spectra was set to 15,000 at m/z 400.

To measure racemization in αA‐crystallin, all forms of the peptide (l‐Asp, l‐isoAsp, d‐Asp and d‐isoAsp) were summed and modifications for each were expressed as a % of the total peak area. The d‐ to l‐form ratio was also calculated according to the peak area in each graph. The ratio was defined as follows: (d‐Asp + d‐isoAsp)/(l‐Asp + l‐isoAsp).

### Statistical analysis

Data are presented as mean values ± standard deviations (S.D.). Statistical analyses were performed using SPSS version 20.0 (SPSS Inc., Chicago, IL, USA). A *P*‐value of <0.05 was considered statistically significant in all cases. Peak areas of specific peptides were calculated using a mean smoothing method. For αA‐crystallin, all forms of the peptide (l‐Asp, l‐isoAsp, d‐Asp and d‐isoAsp) were summed and modifications for each are expressed as a % of the total peak area. Spearman's correlation analysis was used to analyse the correlations between selected variables.

## Results

### Baseline patient characteristics

The baseline characteristics of the HMC and ARC patients enrolled in the study are summarized in Table 1. There were no significant differences between the two groups in terms of age (*P *>* *0.05) and gender(*P *>* *0.05).

**Table 1:** Patient characteristics

Parameter	Age‐related cataract	High myopic cataract
Patients (*n*)	6	6
Mean age (years) ± S.D.	77.8 ± 7.3	75.2 ± 6.4
Gender (Male/Female)	2/4	2/4
Axial length (mm)	22.66 ± 0.71	27.99 ± 1.40

S.D., standard deviation.

### Protein purification and identification of αA‐crystallin

**Figure 1:**
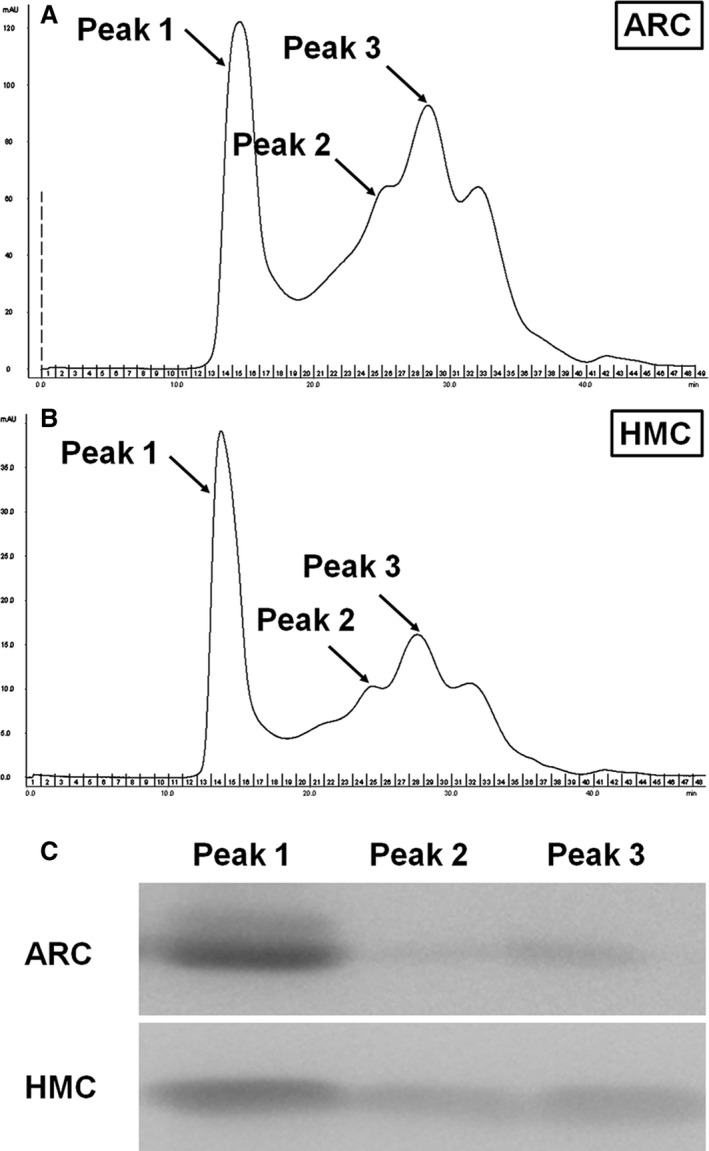
Protein purification and identification of αA‐crystallin. (**A**) Elution profiles of water‐soluble factions of age‐related cataract (ARC) lens proteins using gel filtration chromatography. (**B**) Elution profiles of water‐soluble factions of high myopic cataract (HMC) lens proteins using gel filtration chromatography. All of the lens protein fraction samples of Peak 1, 2 and 3 (arrows) in Fig. 1A and B were collected for further analysis to identify αA‐crystallin. (**C**) Lens protein fraction analysis by Western blotting. The existence of αA‐crystallin was confirmed mainly in Peak 1.

The elution profiles of water‐soluble fractions of ARC and HMC lenses are shown in Figure 1A and B. Three peaks were dominantly observed in the elution profiles (marked Peak 1, 2, and 3 in Figure 1A and B), and these represent the contents of α‐crystallin with high‐molecular weight factions, β‐crystallin and γ‐crystallin with low‐molecular weight fractions according to previous studies 10, 11. Therefore, the lens protein fractions in the three peaks were further analysed to specifically identify αA‐crystallin. Western blot analysis confirmed the existence of αA‐crystallin. In accordance with the previous findings 10, 11, αA‐crystallin was mainly enriched in Peak 1 (Fig. 1C).

### Identification of Asp isomers in αA‐crystallin

**Figure 2:**
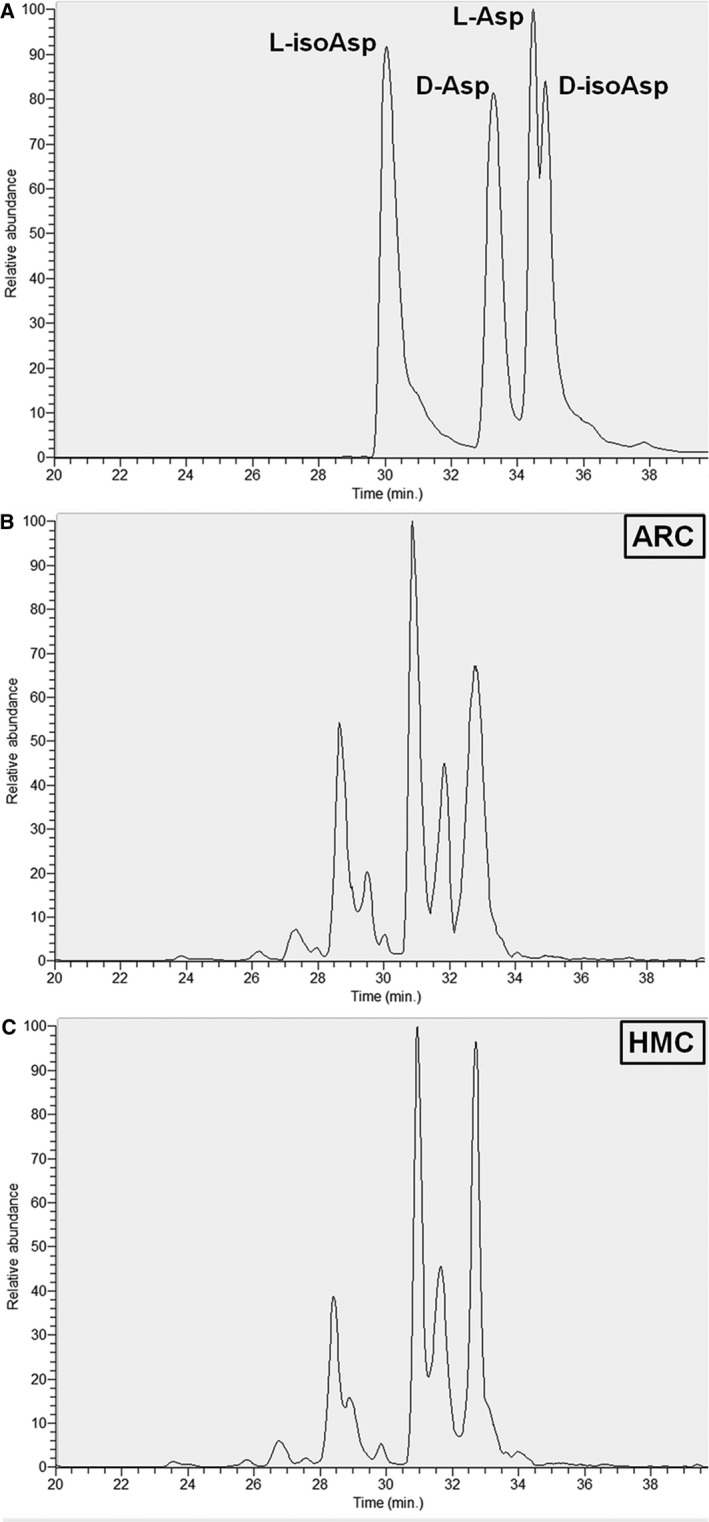
Identification of Asp isomers in αA‐crystallin. (**A**) Representative LC‐MS/MS trace showing the separation of the four Asp isoforms of the αA‐crystallin tryptic peptide (55–65) TVLDSGISEVR. Peptides containing d‐Asp, d‐isoAsp, l‐Asp or l‐isoAsp at position 58 were synthesized. To measure racemization in αA‐crystallin, all forms of the peptide were summed and modifications for each were expressed as a % of the total peak area. (**B**) Representative graphs showing the separation of the four Asp 58 isoforms in αA‐crystallin extracted from age‐related cataract (ARC) lenses. In this ARC case, the ratio of four distinct isomeric forms was l‐isoAsp21.79%, d‐Asp 29.22%, l‐Asp 16.58% and d‐isoAsp 32.40%.(**C**) Representative graphs showing the separation of the four Asp 58 isoforms in αA‐crystallin extracted from high myopic cataract (HMC) lenses. In this HMC case, the ratio of four distinct isomeric forms was l‐isoAsp17.74%, d‐Asp 41.68%, l‐Asp 14.04% and d‐isoAsp 26.54%.

As shown in Figure 2A, an LC‐MS/MS analysis of the four peptide standards (l‐isoAsp, d‐Asp, l‐Asp and d‐isoAsp) by electrospray ionization showed that the intensities of some fragment ions differed between the isoAsp and Asp versions. LC‐MS/MS spectra of the peaks from the human lens digests of ARC and HMC patients (Fig. 2B and C) revealed the same MS/MS spectra as those of the corresponding standards, thus confirming their identification.

### Quantification of Asp racemization in HMC

In our study, no significant correlations were detected between the Asp 58 racemization levels of αA‐crystallin and the age or gender of ARC and HMC patients (*P *>* *0.05).

**Figure 3:**
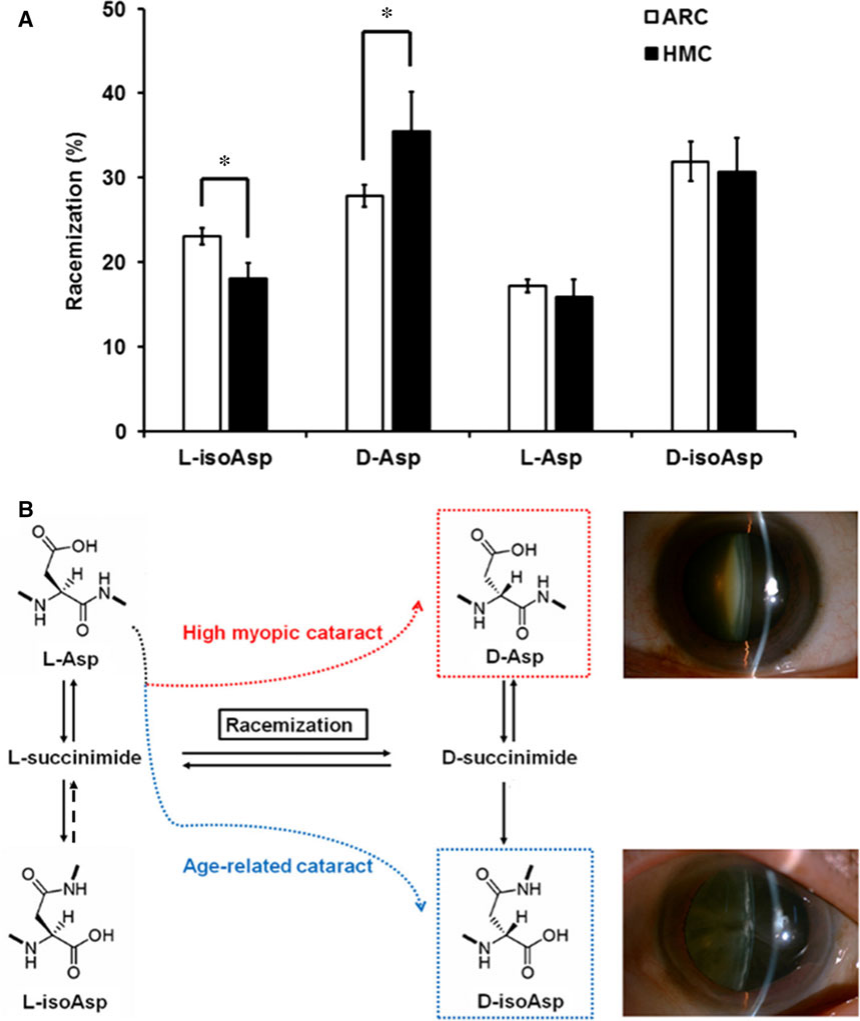
(**A**) Bar diagram showing the amount of Asp 58 racemization in αA‐crystallin of age‐related cataract (ARC) and high myopic cataract (HMC) lenses. Compared with ARC lenses, HMC lenses showed a significant decrease in the amount of l‐isoAsp 58 (*P *<* *0.001) and a significant increase in the amount of d‐Asp 58 (*P *=* *0.009). However, there was no significant difference in the amount of l‐Asp 58 and d‐isoAsp 58 between ARC and HMC lenses (*P *>* *0.05). Thus, in HMC lenses, there appears to have been more conversion of L‐Asp to D‐Asp at the expense of L‐isoAsp. (**B**) Illustration of the normal l‐Asp residues spontaneously converted to l‐isoAsp, d‐Asp and d‐isoAsp isomers. According to our findings, Asp 58 residues in HMC lenses exhibited a different trend in Asp racemization compared to that of ARC lenses. The main difference in racemization at Asp residues of HMC lenses was a greater tendency for the conversion of l‐Asp 58 to d‐Asp 58, instead of to d‐isoAsp 58.

For further analysis, average racemization rates for each Asp isoform were calculated in each group (Fig. 3A). Significantly lower l‐isoAsp (18.05% ± 1.81% *vs*. 23.09% ± 0.98%) and higher d‐Asp 58 (35.44% ± 4.70% *vs*. 27.84% ± 1.33%) levels were identified in αA‐crystallin of HMC lenses than in αA‐crystallin of ARC lenses (*P *<* *0.001 and *P *=* *0.009, respectively). In HMC lenses, it seemed to have been more conversion of l‐Asp to d‐Asp at the expense of l‐isoAsp. The HMC group showed an average d/l ratio of 1.95, which was higher than the ratio in the ARC group of 1.48 (*P *<* *0.001).

Based on our data, we summarized differences in the conversion of racemization in Asp residues of αA‐crystallin in HMC and ARC lenses based on the known spontaneous mechanism of Asp racemization (Fig. 3B). In ARC patients, the conversion of l‐Asp 58 to d‐isoAsp 58, as high as 31.89%, accounted for the main proportion in racemization, which was in accordance with the previous studies conducted by Hooi *et al*. and Aki *et al*. 9, 13. However, in HMC lenses, the conversion of l‐Asp 58 to d‐Asp 58, as high as 35.44%, accounted for the largest proportion of racemizationin αA‐crystallin.

**Figure 4:**
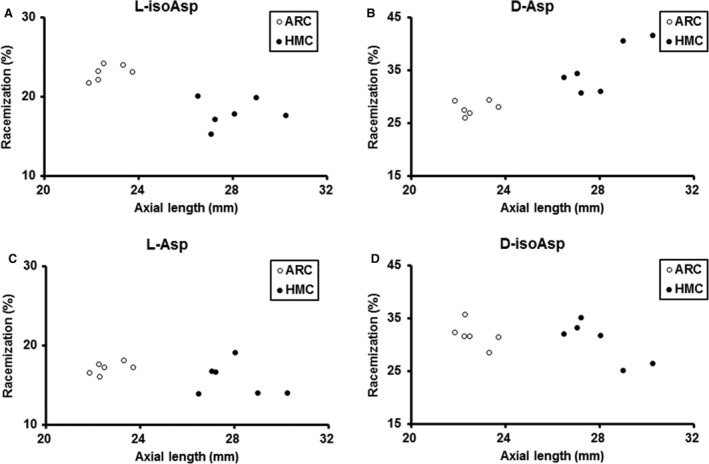
Scatter plot showing the amount of Asp58 racemization in αA‐crystallin for each sample from the age‐related cataract (ARC) and high myopic cataract (HMC) groups. (**A**) Statistical analysis showed a significantly negative correlation between axial lengths and the racemization level of l‐isoAsp 58 (Spearman ρ = −0.699, *P *=* *0.011). (**B**) There was a significantly positive correlation between axial lengths and the racemization level of d‐Asp 58 (Spearman ρ = 0.839, *P *=* *0.001). (**C**) No significant correlation was detected between axial lengths and the amount of l‐Asp 58in ARC and HMC lenses (*P *>* *0.05). (**D**) No significant correlation was detected between axial lengths and the amount of d‐isoAsp 58 in ARC and HMC lenses (*P *>* *0.05).

The overall results for Asp 58 racemization in αA‐crystallin from lenses in the ARC and HMC groups are shown in Figure 4. A significant negative correlation between axial lengths and the racemization level of l‐isoAsp 58 (Spearman ρ = −0.699, *P *=* *0.011) and a significant positive correlation between axial lengths and the racemization level of d‐Asp 58 (Spearman ρ = 0.839, *P *=* *0.001) were identified. No significant correlations were found between axial lengths and the amount of l‐Asp 58 or d‐isoAsp 58 (*P *>* *0.05).

In addition, we further analysed the association between racemization levels and nuclear grading of HMC lenses. We found that HMC lenses with higher nuclear grading, or dark nuclei (NC 4–6 according to the LOCSIII classification), showed a similar racemization level in αA‐crystallin compared to that of lenses with a lower grading (NC 2–3 according to the LOCSIII classification). Based on racemization data from water‐soluble fractions, there was no statistically significant difference in racemization level between HMC lenses with higher nuclear grading and those with lower nuclear grading (*P *>* *0.05, see Fig. S1).

## Discussion

In this study, we examined the racemization levels of three unusual Asp isomers derived from the original l‐Asp at position 58 in the most abundant lens protein, αA‐crystallin, in both ARC and HMC lenses. All three isomers were detected, and the four Asp peptide isoforms were separated. Our findings for ARC lenses were in accordance with previous studies, confirming that the conversion of l‐Asp 58 to d‐isoAsp 58 in αA‐crystallin accounted for the largest proportion of Asp residue racemization 9, 13. However, elevated conversion of l‐Asp 58 to d‐Asp 58, instead of to d‐isoAsp 58, in αA‐crystallin was identified in HMC lenses, indicating that Asp 58 residues in HMC lenses may have undergone a different process of Asp racemization compared to that of ARC lenses. Protein structural changes corresponding to amino acid racemization may affect protein function, which may be related to the intrinsic mechanism of the early onset and different phenotypes of cataract in HMC.

High myopia, defined as an axial length of ≥26 mm 1, is characterized by an early onset of nuclear cataract. Elevated oxidative stress in the vitreous cavity might be responsible for such early cataractogenesis in high myopic patients. With the elongation of axial length, the liquefaction of the vitreous progresses much more rapidly in HMC than in ARC patients 14. Compared to the gel vitreous, the liquid vitreous has a lower concentration of ascorbate and a slower rate of oxygen consumption 15. Therefore, the normal oxygen gradients within the eye are replaced by significantly increased oxygen tension in the vitreous cavity of HMC eyes. Such increased oxygen tension around the lens exposes its proteins to marked oxidative stress, which significantly accelerates the occurrence of PTMs 16 and explains the early cataract formation in high myopia.

Protein misfolding, aggregation and insolubilization can contribute to the onset of age‐related diseases 17, 18, including ARC. It is believed that an important factor that may contribute to protein denaturation in ageing organisms is the intrinsic instability of some amino acid residues. As indicated previously, Asp residues appear to represent ‘hot spots’ in proteins, particularly in unstructured regions, and they are therefore susceptible to modification over time, including racemization. Racemization of Asp in αA‐crystallin of ARC lenses has been extensively studied 6, 9, 19. However, its extent and specific conversion patterns among isomers in HMC remain unknown.

Therefore, we intensively analysed the racemization of Asp 58 in αA‐crystallin of HMC lenses and found a significantly higher racemization level of Asp 58 in the HMC group than in the ARC group (d/l ratio, 1.95 and1.48, respectively). Age‐related changes in Asp 58 may affect the function of αA‐crystallin, as the accumulation of modified Asp residues in proteins may result in conformational changes and protein aggregation 20. Likewise, variation in the levels of racemization in αA‐crystallin of HMC lenses may also lead to the structural transformation of αA‐crystallin, which consequently leads to the abnormal folding and aggregation of proteins, followed by a failure to maintain the normal functioning of heat shock protein, and eventually leads to lens opacities and cataract formation.

In the case of Asp, four distinct isomeric forms (Fig. 3), d‐Asp, d‐isoAsp, l‐isoAsp and l‐Asp, can be spontaneously produced at each site 19. These isomers can have major effects on the protein structure, as different side chain orientations can induce an abnormal peptide backbone. Therefore, the presence of the isomers may be one of the triggers of the partial unfolding of proteins and abnormal aggregation, leading to a disease state. Previous studies, including the studies of Fujii *et al*. and Hooi *et al*., have intensively analysed the racemization of Asp 58 in normal and ARC lenses, and d‐isoAsp 58 seem to be the most dominant d‐isoform. Here, we demonstrated, for the first time, the racemization of Asp 58 in αA‐crystallin isolated from HMC individuals and showed that d‐Asp 58 was the most dominant d‐isoform that accumulated in HMC lenses 21, 22. Considering that the levels of the three isoforms derived from l‐Asp in HMC lenses were significantly different from those in ARC lenses, we inferred that Asp 58 residues in HMC lenses undergo a different pattern of Asp racemization. Compared to the high rate of the cortical type in ARC, HMC patients predominantly present with nuclear cataract characterized by an earlier onset and a more severe lens opacification. Therefore, the significantly increased level of d‐Asp 58 in HMC lenses, different from the previous finding of an increase in d‐isoAsp 58 in age‐matched ARC lenses, may contribute to the distinct pathogenesis and phenotype of cataract in high myopic patients.

In addition, we found that the racemization level of d‐Asp 58 increases with the elongation of axial length in HMC lenses. As previously stated, liquefaction of the vitreous may occur earlier in eyes with longer axial lengths. Therefore, it is possible that HMC patients with longer axial lengths are more likely to maintain a status of excessive oxidation, which may be associated with an increase in racemization. We also identified a similar αA‐crystallin racemization level for the soluble proteins of HMC lenses with different grading. Differences in racemization between lenses with different grading may be seen in the insoluble fraction. However, it is technically difficult to analyse this part as the protein present in the lens centre is mostly coloured, cross‐linked and aggregated, with progressive denaturation 23, 24. On the other hand, the similarity of racemization among high myopic lenses from one aspect proved the difference between HMC and ARC lenses.

Besides, it is also important to mention that as protein‐L‐isoaspartate (D‐aspartate) O‐methyltransferase (PIMT), which is a repair enzyme that initiates the conversion of l‐isoAsp and d‐Asp residues to l‐Asp residues 25, 26, is lacking in the human lens nucleus and no enzyme is present to change either d‐Asp or l‐isoAsp, our results are unlikely to reflect differences in PIMT or any other enzymes in the two groups. Anyway, d d‐isoAsp is not a substrate for PIMT.

## Conclusions

In conclusion, our study revealed that HMC lenses exhibit an elevated racemization of l‐Asp 58 to d‐Asp 58 in αA‐crystallin, rather than from l‐Asp 58 to d‐isoAsp 58, as observed in ARC lenses. The increase in d‐Asp 58 residues in αA‐crystallin of HMC lenses provides a possible explanation for the early onset and different phenotypes of cataract in high myopic patients.

## Ethics approval and consent to participate

All tissues obtained during surgery were handled in accordance with the tenets of the *Declaration of Helsinki*. The Ethics Committees of the Eye and ENT Hospital, Fudan University, Shanghai, China and Guangming Hospital, Jinjiang, Jiangsu, China approved our collection and use of lens tissues from patients undergoing cataract surgery. Informed consent was obtained from all participants. The present investigative study was affiliated to Shanghai High Myopia Study (registered at www.clinicaltrials.gov, accession number NCT03062085).

## Conflict of interests

The authors declare that they have no conflicts of interests.

Consent for publication: Not applicable.

Availability of data and material: All data generated or analysed during this study are included in this published article.

## Supporting information


**Figure S1** High myopic cataract (HMC) lenses with higher nuclear grading, or dark nuclei (NC 4–6 according to the LOCSIII classification), showed a similar racemization level of αA‐crystallin compared to that of lenses with a lower grading (NC 2–3 according to the LOCSIII classification). According to the racemization data from water‐soluble fractions, there was no statistically significant difference in racemization levels between HMC lens with higher nuclear grading and those with lower nuclear grading (*P *>* *0.05).
