# Supplementary figures and images for: Racemization at the Asp 58 residue in αA‐crystallin from the lens of high myopic cataract patients
Source: J Cell Mol Med. 2017 Oct 10;22(2):1118–26. doi: 10.1111/jcmm.13363 (PMC5783843; doi:10.1111/jcmm.13363)

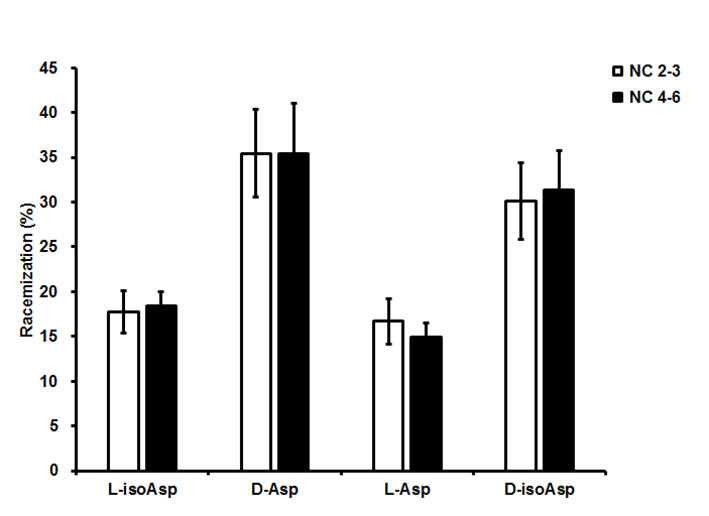

Supplement: Supplementary file 1 — Figure S1 High myopic cataract (HMC) lenses with higher nuclear grading, or dark nuclei (NC 4–6 according to the LOCSIII classification), showed a similar racemization level of αA‐crystallin compared to that of lenses with a lower grading (NC 2–3 according to the LOCSIII classification). According to the racemization data from water‐soluble fractions, there was no statistically significant difference in racemization levels between HMC lens with higher nuclear grading and those with lower nuclear grading (P > 0.05). [file JCMM-22-1118-s001.tif]
